# Tendon compliance and preload must be considered when determining the in vivo force–velocity relationship from the torque–angular velocity relation

**DOI:** 10.1038/s41598-023-33643-9

**Published:** 2023-04-21

**Authors:** Denis Holzer, Matthew Millard, Daniel Hahn, Tobias Siebert, Ansgar Schwirtz, Wolfgang Seiberl

**Affiliations:** 1 Biomechanics in Sports, Department of Sport and Health Sciences, Technical University of Munich, Georg-Brauchle-Ring 60/62, 80992 Munich, Germany; 2 Institute of Engineering and Computational Mechanics, University of Stuttgart, Stuttgart, Germany; 3 Department of Motion and Exercise Science, University of Stuttgart, Stuttgart, Germany; 4 Human Movement Science, Faculty of Sport Science, Ruhr University Bochum, Bochum, Germany; 5 School of Human Movement and Nutrition Sciences, University of Queensland, Brisbane, Australia; 6 Institute of Sport Science, Department of Human Sciences, Universität der Bundeswehr München, Neubiberg, Germany

**Keywords:** Computational science, Biological physics, Tendons, Skeletal muscle

## Abstract

In vivo, the force–velocity relation (F–v–r) is typically derived from the torque–angular velocity relation (T–ω–r), which is subject to two factors that may influence resulting measurements: tendon compliance and preload prior to contraction. The in vivo plantar flexors’ T–ω–r was determined during preloaded maximum voluntary shortening contractions at 0–200°/s. Additionally, we used a two factor block simulation study design to independently analyze the effects of preload and tendon compliance on the resulting T–ω–r. Therefore, we replicated the in vivo experiment using a Hill-type muscle model of the gastrocnemius medialis. The simulation results matched a key pattern observed in our recorded in vivo experimental data: during preloaded contractions, torque output of the muscle was increased when compared with non-preloaded contractions from literature. This effect increased with increasing contraction velocity and can be explained by a rapidly recoiling tendon, allowing the contractile element to contract more slowly, thus developing higher forces compared with non-preloaded contractions. Our simulation results also indicate that a more compliant tendon results in increased ankle joint torques. The simulation and the experimental data clearly show that the deduction of the in vivo F–v–r from the T–ω–r is compromised due to the two factors preloading and tendon compliance.

## Introduction

One of the most fundamental characteristics of skeletal muscle is the force–velocity relation (F–v–r), which has been researched for over a century^1^. From in vitro studies, it is well established that a muscle’s force capacity decreases with increasing shortening velocity. This relation can be described by a rectangular hyperbola^2^. However, by now it is not possible to measure in vivo muscle forces non-invasively, so that the F–v–r of in vivo human muscles is commonly inferred from the joint torque-angular velocity relation (T–ω–r). However, when estimating F–v–r from T–ω–r, a variety of factors such as muscle architecture^3,4^, moment arm geometry^5–7^ and interaction of the contractile and series elastic components^8,9^ need to be considered.

Another factor to consider when obtaining T–ω–r is the level of preload prior to the movement onset^9–13^. Preload is typically applied to minimize the effects of the electromechanical delay, to guarantee maximal muscle activity and to reduce muscle-tendon unit (MTU) compliance. However, preload before movement onset also leads to a stretch of the series elastic component which later can recoil rapidly during shortening. In that case, the recoiling of the stretched elastic tendon allows the fascicles to contract more slowly and develop larger forces according to the fascicles’ F–v–r^8,14^. Thus, preloaded and non-preloaded contractions may result in different T–ω–r characteristics due to tendon compliance. Accordingly, the amount of tendon compliance would therefore also influence estimated in vivo F–v–r, particularly in muscles with a large tendon-to-fascicle-length ratio such as the triceps surae^15^. In vivo, Achilles tendon strain is typically determined by measuring the tendon length change during muscle contractions relative to its slack length by tracking the myotendinous junction displacement relative to the tendon’s insertion point using ultrasound^16^. Tendon compliance is then defined by a single number: the overall strain of the tendon when it is loaded by the maximum isometric force of its contractile element^15–17^. However, Achilles tendon compliance reported in literature varies strongly, ranging from an average maximum strain of 4.9%^18^ up to 9.2%^19^. This wide range might be due to the limitations of this method^16^, and also due to intersubject variability in tendon stiffness. However, a maximum Achilles tendon strain of 4.9%, which is typically used in muscle modeling, seems to be an underestimation, as various studies have found higher strains during dynamic daily activities such as running (3.5–5.8%), walking (4.6%) and one-legged hopping (8.0–8.3%)^20–25^. To our knowledge, the effect of different Achilles tendon compliance on the T–ω–r, and thus in vivo F–v–r estimates, has never been analyzed before.

The aim of this study was to examine the effect of tendon compliance and preload on the T–ω–r of the plantar flexor muscles. We hypothesized that tendon compliance affects muscle shortening velocity leading to differences between preloaded and non-preloaded T–ω–r profiles observed in in vivo studies^12^. As tendon properties cannot be manipulated in vivo, we therefore used a two factor block simulation to independently analyze how tendon compliance and preload affect the resulting T–ω–r of the human plantar flexors. To put our simulation results into context, we compared our simulation results with T–ω–r from literature^26,27^. However, data from preloaded contractions is sparse and shows large variations^26^. Therefore, we also obtained an experimental T–ω–r of the plantar flexors performing preloaded contractions to obtain a more detailed profile.

## Materials and methods

### Sample

Eleven male participants (age: 30 ± 6 years, height: 180 ± 4 cm, mass: 80 ± 4 kg), free of any musculoskeletal and neural impairments in the right lower limb, voluntarily participated in the study after providing their free written informed consent. The experimental protocol was approved by the Faculty of Sport Science Ethics Committee at Ruhr University Bochum in accordance with the Declaration of Helsinki.

### Experimental setup

Participants performed maximum voluntary shortening contractions (MVCs) with their right plantar flexors on a dynamometer (IsoMed2000, D&R Ferstl GmbH, Germany). Participants lay in a prone position with their hip and knee fully extended. The rotational axis of the dynamometer was visually aligned with the right ankle’s axis of rotation at rest. The foot was tightly strapped to a customized footplate attached to the dynamometer’s lever to minimize heel displacement. The right lower limb and upper body were secured by adjustable straps and pads at the shoulders, hip and knee.

**Figure 1:**
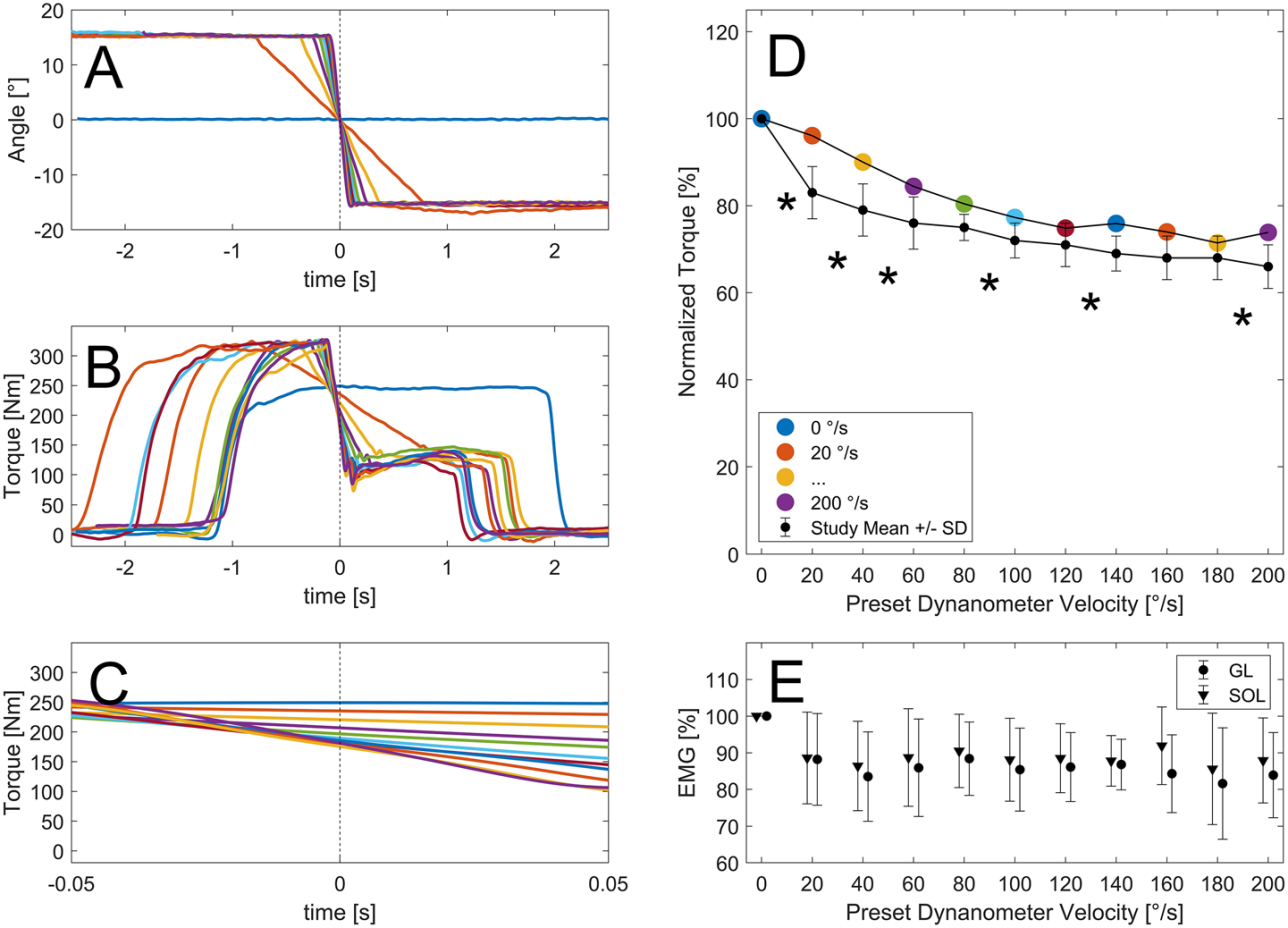
Experimental data for one exemplary participant. (**A**) Maximum voluntary shortening contractions were performed on a dynamometer moving from 15° dorsiflexion to 15° plantar flexion with angular velocities set between 20 and 200°/s with an additional fixed end reference contraction at 0°. (**B**) Dynamometer rotation was automatically initiated when ankle joint torque exceeded 95% of the individual maximum fixed-end plantar flexion torque at 15° dorsiflexion. (**C**) Torque measured when passing through 0° was used to create the individual and mean torque-velocity relationship (**D**). Muscle activity when passing through 0° was unaffected by contraction velocity (**E**). The legend provided in (**D**) serves as the legend for all colored subplots. *Statistically significant difference between successive contraction velocity conditions (p < 0.05).

For the MVCs, dynamometer velocity was preset to ten different angular velocities between 20°/s and 200°/s and performed in a randomized order with a dynamometer crank arm acceleration of 400°/s^2^. All shortening contractions consisted of a fixed-end contraction phase at 15° dorsiflexion, followed by a 30° rotation of the ankle joint, ending in a brief fixed-end contraction phase at 15° plantar flexion which was implemented to ensure that the participants didn’t stop the MVC before the rotation ended (Fig. 1A,B). The neutral ankle joint position (0°) refers to the shank’s longitudinal axis perpendicular to the sole of the foot. During MVCs, ankle joint rotation was triggered by the dynamometer when ankle joint torque exceeded 95% of the individual maximum fixed-end plantar flexion torque at 15° dorsiflexion, which was previously determined during three maximum voluntary fixed-end contractions (MVICs) in the respective ankle joint position (Fig. 1A,B). In addition, two fixed-end MVICs at 0° were randomly interspersed in the measurement sequence. In all shortening conditions the better out of two recorded trials (trial with the higher torque produced at 0°) was used for statistical analysis. The better fixed-end contraction trial was used to normalize the T–ω–r and muscle activities (Fig. 1D,E). The main variable of interest was the torque measured at 0° during the MVCs (Fig. 1C).

### Achilles tendon force estimation

The dynamometer was used to measure the net ankle joint torque during MVCs. Torque was sampled at 1 kHz and synchronized using the Vicon Nexus software (Oxford Metrics, Oxford, UK). Net ankle joint torque was smoothed (4th order dual pass low-pass filter; 10 Hz) and corrected for angle specific gravitational and passive torque using MATLAB (The MathWorks, Inc., Natick, Massachusetts, United States) (see supplementary Material).

M. gastrocnemius medialis (GM) Achilles tendon force was calculated from the net ankle joint torque divided by the individual Achilles tendon moment arm and weighted according to its anatomical proportion of the plantar flexors (15.9% based on the physiological cross-sectional area)^28^. The individual Achilles tendon moment arm was determined by the tendon excursion method^29^ (see Supplementary Material). Alterations of the Achilles tendon moment arm during maximum contractions were considered using a factor of 1.22 as suggested by Maganaris et al.^30^.

### Ankle joint kinematics

Due to soft tissue compression and dynamometer deformation during MVCs, ankle joint angles were determined using a ten-camera motion analysis system at 200 Hz (Vicon Peak, Oxford, UK) synchronized with the dynamometer. Reflective markers (14 mm) on predefined anatomical landmarks (lateral malleolus, medial malleolus, lateral femoral condyle (most lateral aspect), first and fifth metatarsophalangeal joint) defined the foot and shank segments. Marker data was smoothed (4th order zero lag low-pass filter; 6 Hz) using the Vicon Nexus software (Oxford Metrics, Oxford, UK) and then transferred into MATLAB (The MathWorks, Inc., Natick, Massachusetts, United States) for further analysis. Joint angles are relative to the segment angles measured in the standardized static reference position at 0°.

### Surface electromyography (EMG) and ultrasound measures

Muscle activity of the gastrocnemius lateralis, soleus and tibialis anterior muscles was recorded at 1 kHz (OT Bioelettronica, Italy) through adhesive Ag/AgCl surface electrodes (H124SG, CovidienTM, Germany), placed according to the SENIAM recommendations^31^. A single reference electrode was attached onto the left lateral malleolus. EMG data was offset corrected, filtered (4th order bandpass; 10–500 Hz), full-wave rectified and smoothed (75 ms moving average). For the plantar flexors, the peak smoothed EMG signal out of all fixed-end MVCs at 0° served for EMG normalization.

Fascicle length of the right GM was recorded via B-mode ultrasound at 61.5 Hz (Echoblaster 128, UAB Telemed, Lithuania) using a 60 mm linear probe (7 MHz; image depth 50 mm), placed at ~ 50% of GM length over the mid longitudinal axis. A custom-made mount was used to securely attach the ultrasound transducer to the leg, ensuring minimal displacement throughout the measurement session. A voltage signal generated by the ultrasound systems was used to synchronize the ultrasound data with torque, EMG and kinematic data recorded via Nexus (Vicon Peak, Oxford, UK). A custom routine for the image-processing program ImageJ (ImageJ v.1.48; National Institutes of Health, USA) was used to manually determine GM fascicle length over time. The mean slope of the length changes over time was used to determine the mean fascicle velocity during each of the shortening MVCs. For the normalization of fascicle length we used an optimal fascicle length of 4.2 cm^6^.

### Measurement protocol

The experiment consisted of one measurement session (described above) and one familiarization session to get used to the setup at least 2 days prior to the measurement. This session was used to check the participants’ ability to perform good quality MVCs by the principal investigator. Before each experiment, a warm-up consisting of at least ten submaximal concentric, ten submaximal eccentric and three fixed-end MVICs was used to precondition the MTU^32^. All contractions were performed with verbal encouragement from the principal investigator.

### Simulation

**Figure 2:**
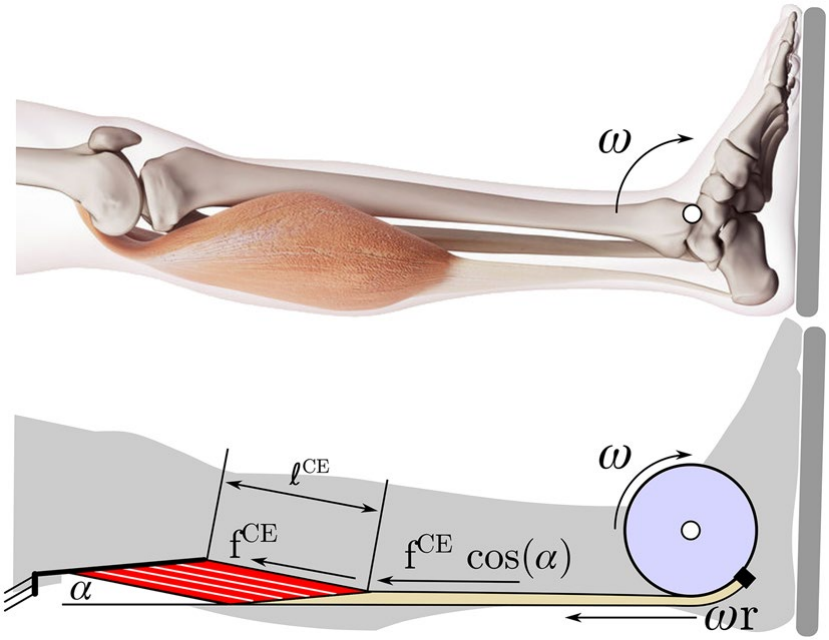
The preloaded and non-preloaded contractions were replicated for the gastrocnemius medialis (GM) by simulation using a Hill-type muscle model^17^. Musculotendon dynamics were simulated as the ankle is moves from 15° of dorsiflexion to 15° of plantar flexion at the prescribed angular velocity. To be consistent with the experimental protocol, samples of the plantar flexor torque, contractile element (CE) length, CE velocity, and tendon velocity were made at 0° of plantar flexion. Since the knee is fixed, we simulated the path of the GM as spanning between a fixed point and terminating at the calcaneus. The Achilles tendon was assumed to have a constant moment arm of radius r. The GM is modelled as a lumped CE of length ℓ^CE^, that acts at an angle α with respect to the tendon (such that ℓ^CE^ sin α is a constant), where the muscle-tendon unit spans a path of length ℓ^P^ between the origin of the CE and calcaneus. As a result, the path velocity of the musculotendon system is v^P^ = ⍵r.

We used a two factor block simulation study design to examine the effect of preload and tendon compliance on the simulated T–ω-r profiles. The experiment was replicated for both the preloaded and non-preloaded protocols by using the Hill-type muscle model of Millard et al.^17^ (which uses a constant thickness pennation model) to simulate the GM (Fig. 2) and its associated Achilles tendon section^33^. We modeled and simulated only the GM, rather than the entire triceps surae, because we were using the model to determine if the factors preload and tendon compliance affect the T–ω–r, rather than trying to precisely match experimental data. The GM model was constructed using architectural parameters from Maganaris^7^ (an optimal contractile element (CE) length (${l}_{o}^{CE}$) of 3.78 cm; a pennation angle ($\alpha$) at ${l}_{o}^{CE}$ of 32.5°; a maximum isometric force (${f}_{o}^{CE}$) of 894.7 N) and from Arnold et al.^16^ (a tendon slack length (${l}_{s}^{T}$) of 40.1 cm). The tendon slack length reported by Arnold et al.^16^ is longer than experimental measurements of the Achilles tendon^34^ because it includes both the length of the Achilles tendon and the full length of the aponeurosis and proximal tendon that connects the contractile element of the GM to its origin. As is typical when simulating younger adults^35,36^, the maximum shortening velocity ${v}_{max}^{CE}$ was set to be ten times the optimal CE length. The CE includes the default nonlinear force–length curve f^L^, passive force f^PE^, and force–velocity curve f^V^, and tendon force–length curve f^T^ that appear in Millard et al.^17^. To examine the effect of tendon compliance on the T–ω–r, we performed all simulations using a rigid tendon, a typical tendon (that develops a modest maximum strain ${e}_{o}^{T}$ of 4.9% at ${f}_{o}^{M}$^18,37^) and a highly compliant tendon (that develops a large maximum strain ${e}_{o}^{T}$ of 9.2% at ${f}_{o}^{M}$^19^). The tendon compliances used should bound any Achilles tendon that is likely to be found in humans. All tendon compliances in between can be expected to result in T–ω–r profiles bounded by the profiles simulated in this study.

Since the knee was fixed during the experiment, we simulated the path length of the muscle as if it spanned between a fixed point (Fig. 1, dashed bar on the left) and the heel. The Achilles tendon moment arm has been fit to the mean value (5.6 cm) of the participants in our experiment (after applying the scale factor of 1.22). We have approximated a constant Achilles tendon moment arm because Rugg et al.'s^38^ measurements indicate that the change in moment arm is negligible (± 3 mm) over the range of motion of the experiment. The origin of the GM model was placed so that the MTU developed its maximum active isometric torque at 17° of dorsiflexion to be consistent with the measurements of Holzer et al.^6^.

As with the experiments, each simulation began with the ankle at 15° of dorsiflexion, and ended when the ankle reached 15° of plantar flexion. For each condition we performed 21 shortening simulations to sample the response of the MTU as the ankle rotated at up to 200°/s in intervals of 10°/s. The T–ω–r profile was constructed by sampling the simulated data at an ankle angle of 0°. We sampled the profiles of the normalized force developed by the MTU (normalized by the static tension during simulated fixed end contractions at 0°), the CE velocity along the tendon,${v}_{AT}^{CE}$ and ${v}^{T}$ the tendon shortening velocity. Under the preload condition, the muscle was initialized and simulated under maximum activation. During the non-preload condition, the muscle was initialized with an activation of zero. As soon as the simulated ankle began to rotate, the muscle was maximally excited and the activation developed according to the first order model described in Millard et al.^17^. We used an activation time constant of 15 ms and a deactivation time constant of 50 ms to be consistent with a typical adult. All numerical simulations were performed using MATLAB^17^.

### Statistics

Normality of distribution and homogeneity of variance were confirmed by the Shapiro–Wilk and Levene’s test (P > 0.05). One-way repeated measures analysis of variance (ANOVA) was performed to identify significant differences in torque, GM MTU force, muscle activity and fascicle length in 0° position across the tested shortening velocities. In case sphericity assumptions were violated, the Greenhouse–Geisser correction was used. With a significant main effect of shortening velocity on GM muscle force, repeated contrasts were analyzed to test for significant differences in force between successive shortening velocities (e.g. 20°/s to 40°/s, 100°/s to 120°/s) to identify the shape of the T–ω–r profile. A Pearson correlation coefficient was used to examine the relationship between angular velocity and mean fascicle shortening velocity. All statistical tests were performed using the Statistical Package for the Social Sciences (IBM SPSS, Chicago, IL, USA) with the level of significance set at α = 0.05.

## Results

### In vivo experiment

**Figure 3:**
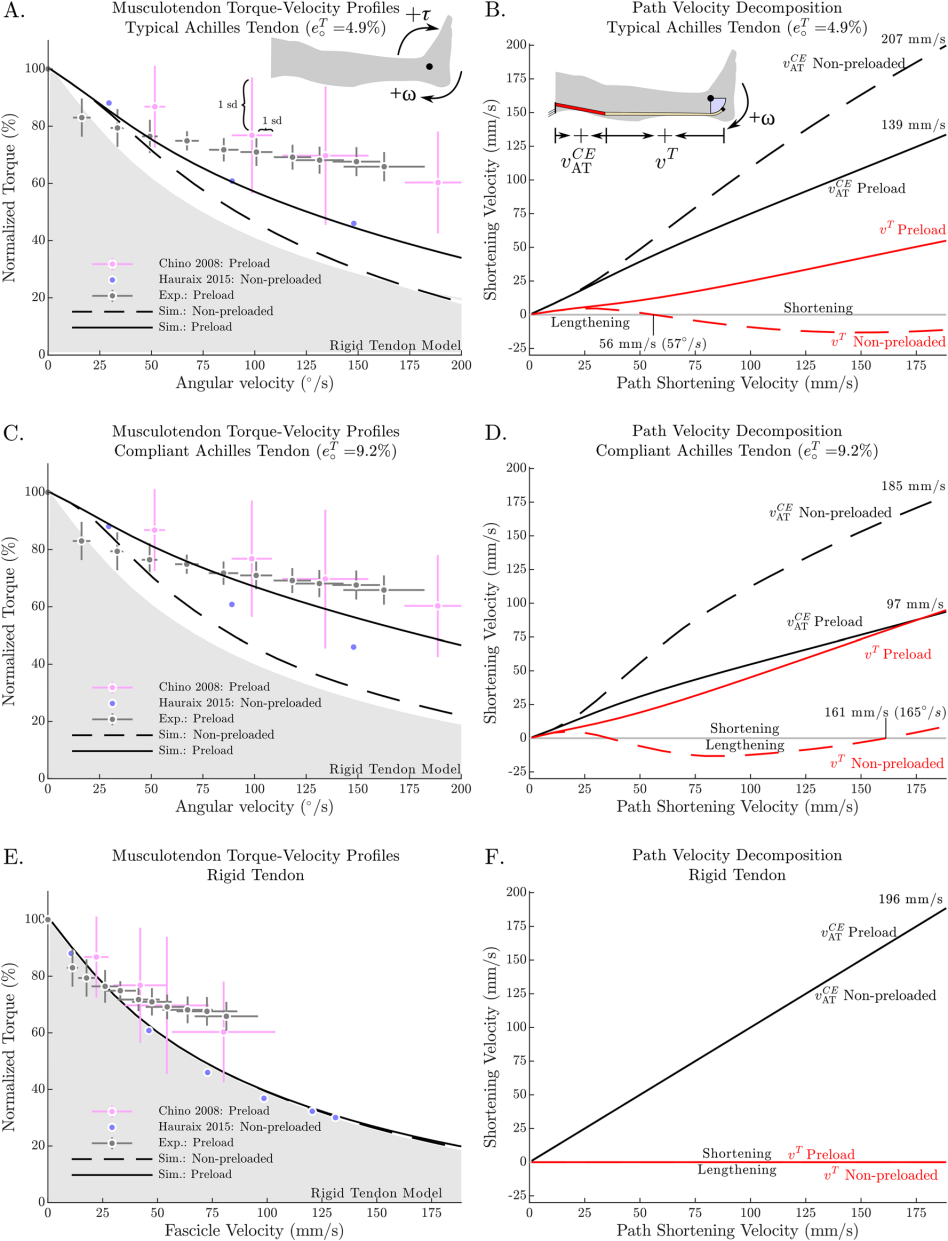
The T–ω–r relationship is influenced by both the amount of preload prior to shortening and the elasticity of the tendon. In the simulation, as the ankle angular velocity increases, a preloaded muscle-tendon unit (MTU) is able to develop larger torques than a non-preloaded MTU (**A**). A similar pattern was found in the experimental data (n = 11), where preloaded protocols (our data and Chino et al.^26^) led to larger torques with increasing angular velocities compared with non-preloaded protocols^27^ as ankle angular velocity increases (**A**). Our simulation results point to the reason for this difference between the two protocols: during a preloaded protocol, the contractile element (CE) shortens more slowly because both, the CE and the tendon shorten together (**B**); in contrast, during a non-preloaded protocol the CE shortens at a higher velocity since the tendon can lengthen when CE force increases. As a result, the force–velocity properties of muscle limit the force of the non-preloaded CE to smaller forces and thus, smaller torques. In the simulation, we found that the difference between the two protocols is more pronounced with a compliant tendon (compare **C** to **A**, and **D** to **B**). In contrast, if a rigid tendon model is used (illustrated as the filled grey area in **A**, **C**, and **E**), there is no difference between the preloaded and non-preloaded protocols (see **E**,**F**). All torques have been normalized to the maximum torque during a fixed-end contraction at an ankle angle of 0°.

For all participants maximal ankle joint torques and GM Achilles tendon forces occurred during fixed-end MVICs at 0° with 218 ± 33 Nm and 630.6 ± 140.3 N, respectively. Repeated measurement ANOVA revealed a significant influence of angular velocity on ankle joint torque and Achilles tendon force (p < 0.001). Thereby, repeated contrasts showed significant differences between adjacent angular dynamometer velocities of 0–20°/s, 20–40°/s, 40–60°/s, 80–100°/s, 120–140°/s and 180–200°/s (p < 0.05). Within the measured angular velocities, mean normalized torque and Achilles tendon force never went below 65% MVIC (Fig. 3A,C).

Pearson’s correlation coefficient revealed a highly positive correlation between ankle joint angular velocity at 0° and mean GM fascicle shortening velocity (R^2^ > 0.99). Repeated measurement ANOVA revealed no significant differences in gastrocnemius lateralis and soleus muscle activity between the tested conditions. The same was true for the controlled antagonistic tibialis anterior muscle activity, where statistics did not identify any differences throughout the tested conditions. For more information please see the table provided in the Supplementary Materials. GM fascicle length at 0° was unaffected by shortening velocity (p = 0.161) (Fig. 5B). Mean calculated Achilles tendon moment arm was 56.0 ± 5.7 mm with individual moment arms ranging from 46.0 to 65.3 mm.

### Model experiments

The simulation of preloaded vs. non-preloaded contractions shows some of the same patterns (Fig. 3A) that appear in in vivo measurements: preloading produces higher torques across the T–ω–r profile^26^ than a non-preloaded protocol^27^. When the MTU is preloaded, both the CE and the tendon shorten together (Fig. 3B), and so, the shortening velocity of the CE is lower than that of the entire MTU. In contrast, during the non-preloaded contractions, the tendon is either at a constant length or is lengthening (Fig. 3B). With a more compliant tendon, the preloaded T–ω–r shows even higher torques at higher shortening velocities (Fig. 3C) because the CE is able to contract more slowly (Fig. 3D). According to our simulations, the differences between the preloaded and non-preloaded contractions are unequivocally due to the compliance of the tendon: the difference disappears when the simulation is repeated using a rigid-tendon (Fig. 3E,F). Tendon compliance also had a slight effect on the simulated CE length at 0^o^ (Fig. 5B,D), with faster shortening velocities being associated with longer CE lengths.

**Figure 4:**
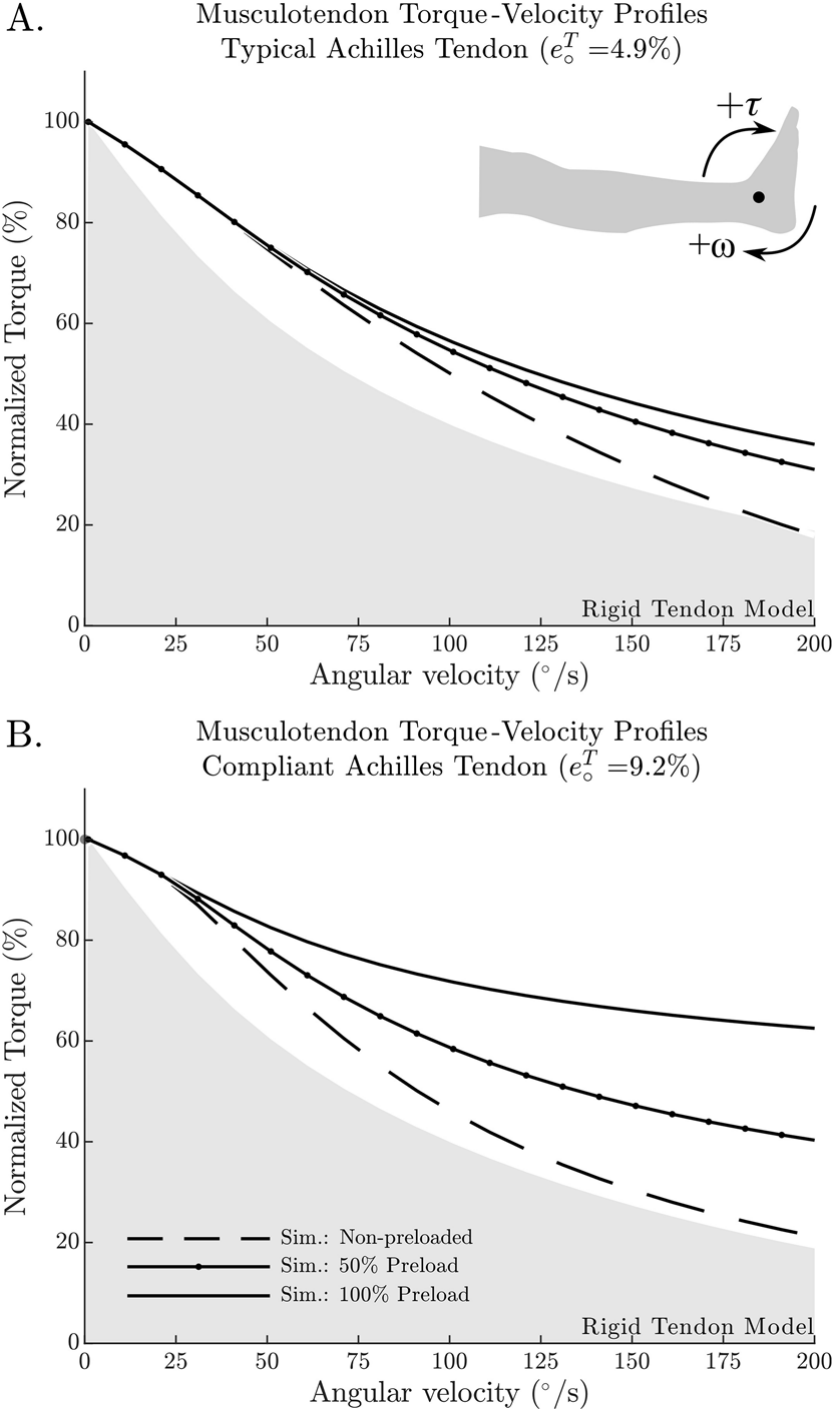
The amount of preload on the Achilles tendon has a nonlinear effect on the resulting T–ω–r during shortening. For example, when shortening is simulated using a 50% preloaded tendon, the resulting T–ω–r profile is not equal to the geometric mean of the non-preloaded and 100% preloaded T–ω–r profiles (**A**). The compliance of the tendon directly affects the relative position of the 50% preloaded simulation: when a typical Achilles tendon is used, the 50% preloaded T–ω–r is closer to the 100% preloaded T–ω–r (**A**); in contrast, when a compliant Achilles tendon is used (**B**), the 50% preloaded T–ω–r is closer to the non-preloaded T–ω–r. As a reference, the output of the rigid tendon model is illustrated as the filled grey area (**A** and **B**).

As would be expected, preloading the MTU by 50% of MVIC yields a T–ω–r that is between the preloaded and non-preloaded results (Fig. 4A). Since both the CE and tendon elasticity are highly nonlinear in a Hill-type muscle model^17^, both preload (Fig. 4A) and tendon elasticity nonlinearly affect the T–ω–r (Fig. 4B).

## Discussion

In the present study, we aimed to investigate the influence of preload and tendon compliance on estimated F–v–r by collecting T–ω–r data from a MTU with a long tendon-to-fascicle-length ratio, and by using a muscle-driven dynamic simulation of the MTU. The results confirm our hypothesis that tendon compliance is responsible for the observed experimental difference between preloaded and non-preloaded T–ω–r profiles.

### In vivo data

Ankle joint torques (218 ± 33 Nm) measured during fixed-end MVICs at 0° were in line with previous literature^6,39^. With increasing shortening velocities, yet constant muscle activity, active ankle joint torques, and estimated GM muscle forces, decreased. The shape of the resulting estimated F–v–r could be described as a hyperbola known from F–v–r experiments on isolated muscles. However, there was a large difference between the common hyperbolic shape of the F–v–r^2^ and our results: ankle torques, and thus estimated muscle forces, were not approaching zero, but rather seemed to plateau at approximately 65% MVIC within the range of measured velocities (Fig. 3A,C). This shape was different from the estimated F–v–r shape reported for non-preloaded contractions of the plantar flexors^27^. The few velocities tested by Chino et al.^26^ led to a comparable normalized ankle joint torque output as observed in this study (Fig. 3A,C). Increased torque output due to preload has previously been shown for the knee joint^12,13,40,41^. However, this is the first study reporting an almost complete “flattening” of the T–ω–r. We think that this is due to the much greater tendon-to-muscle length ratio of the plantar flexors compared with the knee extensors, leading to a greater influence of the tendon on the torque output of the MTU during preloaded shortening contractions.

**Figure 5:**
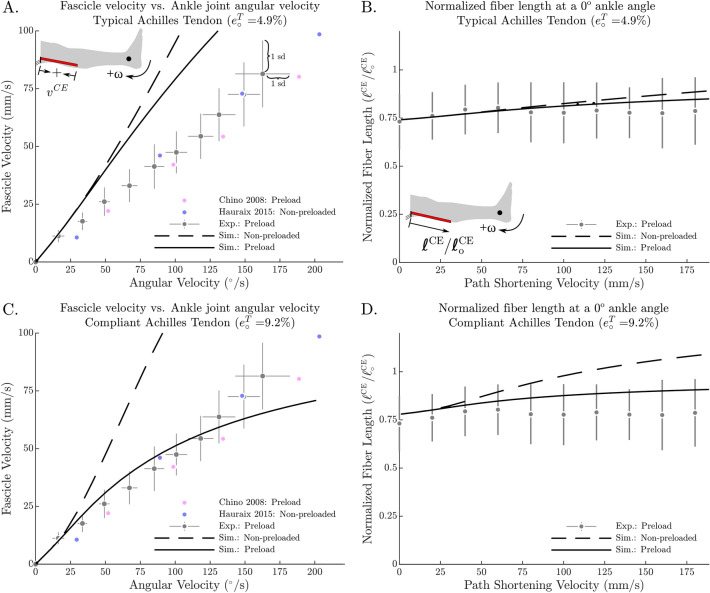
All experimental data show a linear relationship between mean fascicle velocity and ankle angular velocity (**A**,**C**). However, our simulation results indicate that this relationship might be dependent on tendon compliance and preloading since a less compliant (typical) Achilles tendon confirmed this approximately linear relationship between contractile element (CE) velocity and ankle angular velocity at 0° (**A**) whereas a more compliant tendon led to a quite nonlinear relationship with the preloaded protocol (**C**). CE fascicle length at 0° was linear in the experimental data of the current study (**B**). For a typical tendon the simulated normalized CE length was within ± 1 standard deviation (sd) of the in vivo normalized fascicle length measurements for both preloading conditions (**B**). Simulated normalized CE fascicle length at 0° increased noticeably with increasing ankle angular velocity for the compliant tendon (**D**).

From in vitro experiments on isolated muscle fibers, and muscles, it is well established that increasing shortening velocities reduce muscle force capacity. Our results show that mean GM fascicle shortening velocity increased linearly with increasing ankle angular velocity (Fig. 5A), which is in line with the literature^26,27,42,43^. Fontana et al.^12^ have suggested that preloading the MTU stretches the Achilles tendon and allows the fascicles to contract more slowly during the subsequent shortening. This effect would explain the flattening of the T–ω–r. However, we were not able to show such an interaction of muscle and tendon in our in vivo data. This is because accurate tracking of fascicle length changes at very high spatio-temporal resolution would be needed for a numerical derivative to evaluate instantaneous fascicle velocities. Due to the rather low ultrasound recording frequency, however, this was not possible and we present mean velocity instead.

Our results show that experiments with preloaded and non-preloaded contractions both have limitations. Especially in joints with a limited range of motion, such as the ankle joint, non-preloaded contractions only allow submaximal forces as there is not enough time to reach close to maximal muscle forces (~ 300–500 ms^44–46^) especially at high shortening velocities^13^. To account for this problem, preloaded contractions can be used where the muscle is fully activated before the joint rotation is triggered^11–13,26^. This eliminates the problem of incomplete muscle activation, regardless of range of motion and shortening velocity. However, the effect of tendon recoil will lead to an overestimation of fascicle velocity which results in an apparent overestimation in force during high shortening velocities which is in line with data previously reported for the knee extensor muscles^12^. As a result, neither the recorded T–ω–r of a preloaded nor non-preloaded protocol would accurately reflect the F–v–r of the CE.

### Muscle-simulation

The simulation of the experiment was able to reproduce some of the differences observed experimentally between preloaded and non-preloaded contractions (Fig. 3A): preloading results in a T–ω–r profile that shows higher torques than the T–ω–r profile produced by a non-preloaded MTU. The simulation results are consistent with our hypothesis that tendon compliance is responsible for the measured differences between preloaded and non-preloaded T–ω–r profiles (Fig. 3): when preloaded, a compliant tendon allows the CE to contract more slowly (Fig. 3); when non-preloaded, a complaint tendon may result in the CE contracting more rapidly as it stretches the tendon.

The model clearly showed that the difference between the MTU's shortening velocity and that of the CE is affected by different levels of preload, and tendon compliance. Further, we found an interaction between tendon compliance and preload that allowed the fascicles to contract more slowly, ultimately resulting in an increased force potential and thus, higher ankle torques. The simulation of an additional preloading condition with 50% preload confirmed how complex and non-trivial this interaction between tendon compliance and preload is, as the relative torque output between 50% preloaded T–ω–r and the 100% preloaded T–ω–r changed with tendon compliance (Fig. 4). Even though this is not part of the results, it must be noted that the T–ω–r changes depend on the point of measurement (0° ankle angle in the current study) which is in line with in vivo data^13^. In the simulation, the T–ω–r changes with the point of measurement because the CE length changes with force (Fig. 5B,D), and additionally because of the rapidly changing CE shortening velocity during the non-preloaded protocol. To conclude, the observed ankle T–ω–r is highly influenced by the experimental conditions and the mechanical properties of the Achilles tendon.

The simulations indicate that there are a number of secondary factors that will influence the measured torque beyond the shortening velocity of the CE. The model showed that CE length at 0° is not constant in either protocol (Fig. 5B), and that CE length at 0° varies more as tendon compliance increases (Fig. 5D). According to the F–v–r, a faster shortening velocity results in reduced force pulling on the Achilles tendon when passing through 0°. Therefore, at a constant MTU length, the Achilles tendon length decreases with increasing shortening velocities, as there is less force pulling on the tendon. In turn, the CE is operating at a longer length which is beneficial regarding the force–length relationship of the triceps surae in this joint angular position, since the muscle is operating on the ascending limb of its force–length relation^6,7^. Additionally, the total work done by the CE to stretch the tendon and rotate the ankle to the target angle of 0° differed substantially between the different contraction conditions. The non-preloaded contractions with a less compliant tendon produced the least CE work (11.1 J at 0°/s and 1.4 J at 200°/s), while the preloaded contractions and highly compliant tendon resulted in the most CE work (14.5 J at 0°/s and 10.0 J at 200°/s). Since CE work is known to affect the amount of force depression^47,48^, the simulation results suggest that we can expect force depression related effects to further influence the force production during preloaded and non-preloaded contractions.

It must be noted that the clear difference in simulated CE shortening velocity between different preloading protocols is in contradiction to in vivo experiments on the knee extensor muscles^12^, where no significant changes in fascicle shortening velocity were reported between different preload conditions. We believe that the knee extensors with their relatively short tendon-to-fascicle-length ratio will be less affected by tendon compliance than the plantar flexors which have a large tendon-to-fascicle-length ratio^15^. Variations of tendon-to-fascicle-length ratio have been demonstrated to strongly affect contraction dynamics and force generation^49^. Thus, we expected differences regarding fascicle shortening velocity between the different contraction conditions. Yet, the mean fascicle shortening velocities reported by Hauraix et al.^27^ (non-preloaded) were within the standard deviation of the mean fascicle shortening velocities measured in the current study (preloaded) (Fig. 5A). Therefore, we cannot necessarily confirm the simulation results in an in vivo setting. However, this again might be due to our ability to only report mean fascicle shortening speeds instead of angle dependent velocities.

**Figure 6:**
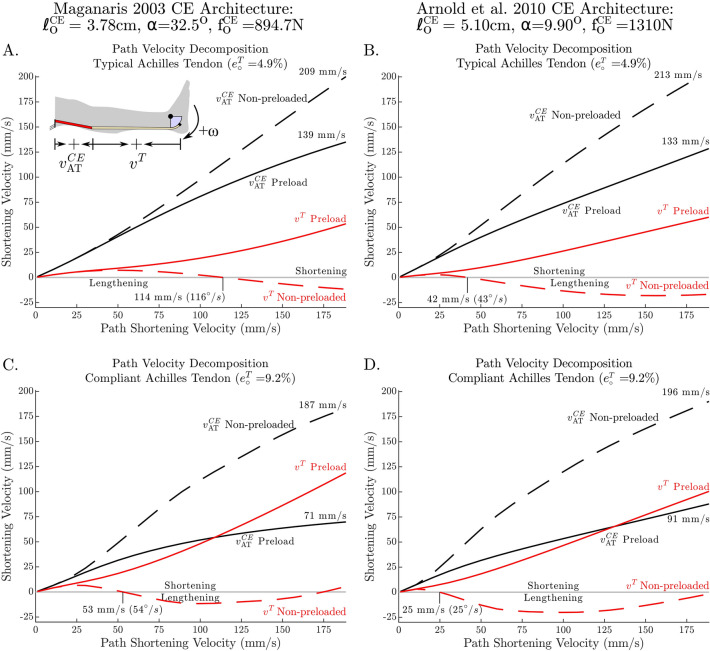
The effects of preload and tendon compliance on simulated T–ω–r shape are robust against changes in the architectural properties of the contractile element (CE). Simulations produced using CE architectural data from Maganaris^7^ (${\mathrm{l}}_{\mathrm{o}}^{\mathrm{CE}}$ of 3.78 cm, $\mathrm{\alpha }$ of 32.5°, and ${\mathrm{f}}_{\mathrm{o}}^{\mathrm{CE}}$ of 894.7 N) and Arnold et al.^16^ (${\mathrm{l}}_{\mathrm{o}}^{\mathrm{CE}}$ of 5.1 cm, $\mathrm{\alpha }$ of 9.9°, and ${\mathrm{f}}_{\mathrm{o}}^{\mathrm{CE}}$ of 1308 N) produce qualitatively similar velocity distributions though the numerical values differ (compare **A** to **B**, and **C** to **D**). Since both sets of results show great differences between the typical (less compliant) and more compliant tendon (compare **A** to **C** and **B** to **D**) it is clear that the properties of the tendon are more influential in this scenario than the architectural properties of the CE.

Although the model was able to reproduce some of the characteristic differences between the preloaded and non-preloaded contractions, there remains a qualitative difference between the simulated and in vivo data of how fascicle velocity varies with ankle angular velocity at 0° (Fig. 5A,C). To see whether the architectural properties of the CE influenced our results, we re-ran the simulation using the CE architectural properties from Arnold et al.^16^ (${l}_{o}^{CE}$ of 5.1 cm, $\alpha$ of 9.9°, and ${f}_{o}^{CE}$ of 1308 N) which differ substantially from the in vivo architectural properties measured by Maganaris 2003 (${l}_{o}^{CE}$ of 3.78 cm, $\alpha$ of 32.5°, and ${f}_{o}^{CE}$ of 894.7 N). While the numerical values produced by these two simulations differ, qualitatively both sets of simulations are similar (in Fig. 6 compare A–B, and C–D). Further, we explored the effects of adding damping to the tendon model using the data of Netti et al.^50^ to derive a model of tendon damping that scales with tendon stiffness $\beta { }^{T}=0.05 K{ }^{T}(\mathcal{l}{ }^{T})$, where $\beta { }^{T}$ is the damping coefficient of the tendon, $K{ }^{T}(\mathcal{l}{ }^{T})$ is the stiffness of the tendon. We found that tendon damping makes the CE velocity vs. ankle angular velocity characteristic fit the data better for the preloaded simulations using the highly compliant tendon, but has little effect on the less compliant tendon. In either case, the addition of tendon damping had little effect on our results since the distribution of tendon and fiber velocities were not affected much (for a plot of these effects see supplementary material). The steepness of the slack simulations (Fig. 5A,C) seems only to be affected by preload. The experiments of Hauraix et al.^27^ were passively preloaded due to the dorsiflexed starting position, and so, perhaps that explains why their characteristic (Fig. 5A,C) agrees more closely with the preloaded experimental data than with our simulations of the non-preloaded protocol.

### Limitations

Co-contraction by the main antagonist (tibialis anterior) was controlled via EMG but not taken into account for the muscle force calculation. However, this unaccounted factor does not influence the analyzed F–v–r shape, as no significant differences in TA activity were detected for the different velocities tested. Additionally, Raiteri et al.^51^ showed only negligible effects of co-contraction during fixed-end plantar flexions. Further, we assumed a constant GM contribution (15.9%) to the total plantar flexion torque. This might be an over simplistic model, as there are eight more plantar flexors, some with their own tendons, contributing to the overall plantar flexion torque.

The in vivo data of this study was obtained from male participants only. Previous investigations have demonstrated sex differences in mechanical properties of the triceps surae muscle–tendon unit, indicating that females tend to generate less muscle and tendon forces, exhibit shorter tendon lengths and smaller cross-sectional areas, and demonstrate more compliant tendons with a lower Youngs’s modulus compared with their male counterparts^52–56^. Therefore, the extent to which the in vivo F–v–r differs from the T–ω–r is likely to be influenced by sex. However, the main finding of a compromised transferability of T–ω–r to in vivo F–v–r properties should hold true regardless of potential sex differences in MTU properties. Since individual differences have been shown to be greater than sex differences, further studies should focus on participant-specific tendon parameters rather than sex specific mean values^57^.

For the computer model, we assumed a constant Achilles tendon moment arm throughout the entire tested range of motion. However, most studies found an ankle angle dependent Achilles tendon moment arm^6^. Again, this should only have slight effects on our main findings as all measurements refer to the 0° position where the moment arm should be constant throughout all velocities.

In contrast to the simulation results, a change in fascicle operating length at 0° was not visible in the ultrasound data of the current study (Fig. 5B,D, for further information see supplementary material). However, this lack of statistically significant differences might be due to a limited sampling frequency and lack of precision of fascicle length measurements using ultrasound making it difficult to find significant differences. Yet, simulation data and in vivo measurements of normalized fascicle length at 0° did match for the different velocities (Fig. 5B,D).

## Conclusion

This study showed that the contraction condition (preloaded versus non-preloaded) drastically affects the shape of in vivo T–ω–r for MTUs with relatively long compliant tendons. Preloading the MTU prior to shortening contractions allows the MTU to produce large forces even at high angular velocities. The simulation of the experiment using a Hill-type muscle model showed that the preloaded contractions resulted in the tendon rapidly recoiling, allowing the CE to contract more slowly and develop higher forces compared with non-preloaded contractions. In contrast, during the non-preloaded contractions, the Achilles tendon was lengthening during the shortening contractions, resulting in the CE (along the tendon) contracting faster than the entire MTU, and thus developing lower forces. The simulation and the experimental data clearly show that the deduction of the in vivo F–v–r from the T–ω–r is compromised due to the factors preload and tendon compliance. Therefore, future studies investigating the in vivo F–v–r of muscles with long compliant tendons should account for tendon compliance. Unfortunately, it is currently very challenging to accurately measure the length of the Achilles tendon during dynamic movement because the location of both ends need to be tracked during the experiment. Alternatively, additional measurements can be made so that the active-force–length, passive-force–length, and tendon elasticity are taken into account when analyzing force–velocity data. Since the elastic tendon allows all properties of the model to affect the force developed by the CE, additional experimental trials should be completed to accurately fit the model: the active and passive fixed-end force–length relation should be measured in addition to the T–ω–r. If the kinematics of the tendon cannot be tracked, then the elasticity of the tendon can be left as a free parameter to be identified by minimizing the sum of squared errors between the experiments and simulations of the experiments using the fitted muscle model.

## Supplementary Information

Supplementary Information.

## Data Availability

The datasets generated during and/or analyzed during the current study are available from the corresponding author on reasonable request. The code to run the simulations of the experiments is implemented in a series of MATLAB scripts available here: https://github.com/mjhmilla/PlantarFlexorDynamometrySimulation.
