# Supplementary material for: Tendon compliance and preload must be considered when determining the in vivo force–velocity relationship from the torque–angular velocity relation
Source: Sci Rep. 2023 Apr 21;13:6588. doi: 10.1038/s41598-023-33643-9 (PMC10121672; doi:10.1038/s41598-023-33643-9)
Supplement: Supplementary file 1 — Supplementary Information. [file 41598_2023_33643_MOESM1_ESM.pdf]

Supplementary information on methods

Tendon compliance and preload must be considered when determining the *in vivo* force-velocity relationship from the torque-angular velocity relation

by Denis Holzer, Matthew Millar[2], Daniel Hahn, Tobias Siebert, Ansgar Schwirtz, Wolfgang Seiberl

Determination of the Achilles tendon moment arm

The individual Achilles tendon moment arm was determined by the tendon excursion method [1]. The right ankle joint was slowly rotated at 5°/s between 5° dorsiflexion and 20° plantar flexion. For this passive ankle joint rotation, the ultrasound probe was moved to the distal end of the gastrocnemius medialis to get a clear image of the myotendinous junction. Within the tested range of motion, tendon deformation due to ankle angle change has shown to be low [2]. Electromyography (EMG) was used to confirm that no muscle activity was present during passive ankle joint rotations. Passive rotations were rejected when the EMG signal was greater than the corresponding resting conditions plus two standard deviations. The myotendinous junction of the gastrocnemius medialis was manually traced using ImageJ (ImageJ v.1.48; National Institutes of Health, USA). The mean slope of linear regression of the tendon excursion versus the ankle joint angular changes (measured via motion tracking) gave an estimation of the individual Achilles tendon moment arm (average of five trials). Alterations of the Achilles tendon moment arm during maximum contractions were considered using a factor of 1.18 suggested by Maganaris et al. [3].

Torque correction

Torques were corrected for angle specific gravitational torque. For this, the participant’s foot was slowly rotated (5°/s) by the dynamometer throughout the tested range of motion (three times in each direction) with the EMG system controlling for any muscular activity. Thereby, passive rotations were invalid/repeated when the EMG signal during a passive rotation of any tested muscle was greater than the EMG signal of the corresponding muscle during resting conditions plus two standard deviations. The mean angle specific passive torque was then subtracted from the corresponding angle specific torque throughout each trial [4,5].

Literature data

Throughout this study, we refer to fascicle contraction velocity, ankle joint angular velocity and Achilles tendon force data published by Hauraix et al., [6] and Chino et al., [7]. If the data were not directly available from text or tables, but presented in graphs, ImageJ was used to estimate mean and standard deviation of the results. Hauraix et al. [6] did not report isometric contractions which are needed for the normalization of the force-velocity data. Therefore, the zero velocity condition was estimated by the hyperbolic equation fitted across the mean force-velocity measurements (6; Figure 2).

Plot of the effects of adding tendon damping to the model

The agreement between the simulated and experimental CE velocity vs. ankle angular velocity characteristic can be improved by adding damping to the tendon element. The improvement is small for a tendon with a typical stiffness (A) but quite pronounced for a more compliant tendon (C). However, the distribution of the path velocity between the tendon and CE is not affected much (B & D): the preloaded protocol still produces lower CE velocities than the non-preloaded protocol.

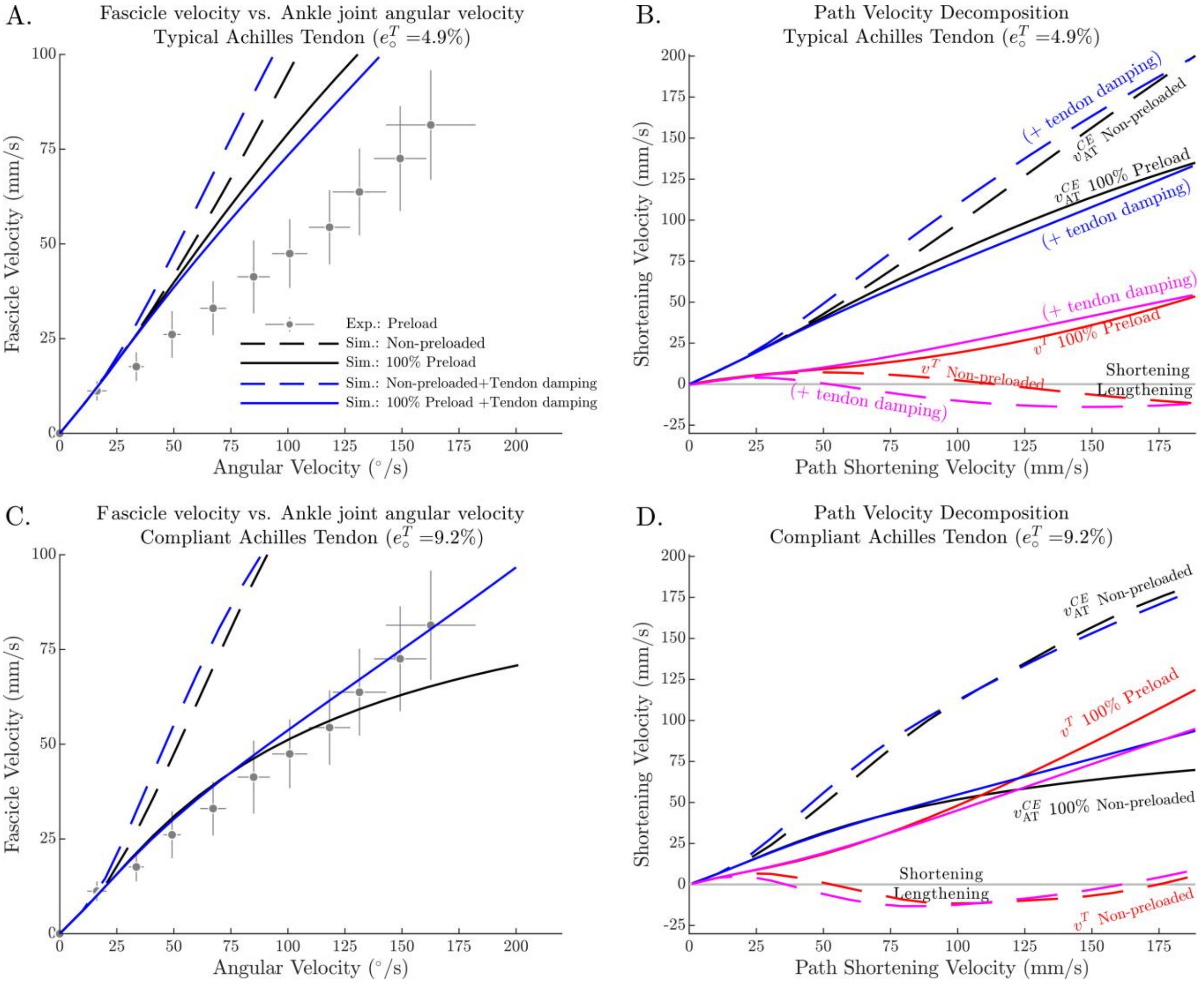

Table 1**:** Discrete parameters for the maximum voluntary shortening contractions at various velocities. All results are presented as mean ± standard deviation and refer to the 0° condition (ankle joint passing through neutral position). With the ANOVA (bottom row) revealing a significant ($p<0.05$) main effect of contraction velocity on a parameter, repeated contrasts were analyzed. *Statistically significant difference to successive contraction velocity condition ($p<0.05$).

**Preset dynamometer velocity [°/s]**	**Measured dynamometer velocity [°/s]**	**Ankle joint angular velocity [°/s]**	**Mean GM fascicle velocity [mm/s]**	**Torque [Nm]**	**GM Achilles tendon force [N]**	**Normalized GM muscle force [%]**	**GL activity [%]**	**SOL activity [%]**	**GM fascicle length [mm]**
**0**	0 ± 0*	0 ± 0*	0 ± 0*	218.4 ± 32.6*	630.5 ± 140.3*	100 ± 0*	100 ± 0	100 ± 0	30.7 ± 6.0
**20**	16.9 ± 1.7*	16.3 ± 4.1*	11.2 ± 2.5*	182.1 ± 34.0*	528.2 ± 145.9*	83.0 ± 6.4*	88.2 ± 12.5	88.6 ± 12.2	32.0 ± 5.2
**40**	36.2 ± 1.5*	33.5 ± 3.2*	17.6 ± 3.6*	173.9 ± 31.4*	504.7 ± 136.8*	79.4 ± 6.3*	83.5 ± 12.2	86.4 ± 17.4	33.3 ± 5.4
**60**	57.1 ± 1.2*	49.2 ± 3.6*	26.1 ± 5.9*	167.4 ± 29.6	485.5 ± 128.5	76.4 ± 5.5	85.9 ± 13.3	88.7 ± 14.1	33.7 ± 5.5
**80**	78.2 ± 1.9*	67.2 ± 5.4*	33.0 ± 6.8*	163.5 ± 24.6*	573.1 ± 111.6*	74.9 ± 3.2*	88.4 ± 10.0	90.5 ± 12	32.7 ± 6.5
**100**	99.0 ± 1.3*	85.0 ± 6.8*	41.3 ± 9.2*	156.6 ± 24.1	453.6 ± 109.7	71.7 ± 3.9	85.4 ± 11.3	88.1 ± 14.2	32.6 ± 6.7
**120**	116.5 ± 2.0*	100.8 ± 7.4*	47.4 ± 8.6*	154.8 ± 24.1*	448.5 ± 109.2*	71.0 ± 4.6*	86.1 ± 9.4	88.5 ± 15.1	33.1 ± 6.1
**140**	133.8 ± 2.7*	118.3 ± 8.6*	54.4 ± 9.4*	151.2 ± 25.1	438.2 ± 112.2	69.2 ± 4.2	86.8 ± 6.9	87.8 ± 9.1	32.7 ± 5.5
**160**	157.5 ± 3.9*	131.4 ± 11.2	63.7 ± 10.9*	149.1 ± 26.2	432.7 ± 115.3	68.1 ± 4.5	84.3 ± 10.6	91.9 ± 10.4	32.6 ± 7.7
**180**	181.4 ± 3.1*	149.2 ± 11.0*	72.5 ± 13.2*	148.0 ± 26.2*	428.5 ± 110.8*	67.6 ± 4.8*	81.6 ± 15.2	85.6 ± 12.2	33.0 ± 7.4
**200**	206.8 ± 2.4	162.3 ± 18.7	81.4 ± 13.8	144.0 ± 25.4	418.1 ± 112.2	65.8 ± 4.9	83.9 ± 11.6	87.9 ± 18.6	32.9 ± 7.8
**ANOVA**	0.000	0.000	0.000	0.000	0.000	0.000	0.137	0.731	0.161

References

1. An, K. N., Ueba, Y., Chao, E. Y., Cooney, W. P. & Linscheid, R. L. Tendon excursion and moment arm of index finger muscles. *Journal of biomechanics* **16,** 419–425 (1983).
2. Monte, G. de, Arampatzis, A., Stogiannari, C. & Karamanidis, K. In vivo motion transmission in the inactive gastrocnemius medialis muscle-tendon unit during ankle and knee joint rotation. *Journal of Electromyography and Kinesiology* **16,** 413–422 (2006).
3. Maganaris, C. N., Baltzopoulos, V. & Sargeant, A. J. Changes in Achilles tendon moment arm from rest to maximum isometric plantarflexion: in vivo observations in man. *The Journal of Physiology* **510,** 977–985 (1998).
4. Bakenecker, P., Raiteri, B. J. & Hahn, D. Force enhancement in the human vastus lateralis is muscle-length-dependent following stretch but not during stretch. *European journal of applied physiology* **120,** 2597–2610 (2020).
5. Holzer, D., Paternoster, F. K., Hahn, D., Siebert, T. & Seiberl, W. Considerations on the human Achilles tendon moment arm for in vivo triceps surae muscle-tendon unit force estimates. *Scientific reports* **10,** 19559 (2020).
6. Hauraix, H., Nordez, A., Guilhem, G., Rabita, G. & Dorel, S. In vivo maximal fascicle-shortening velocity during plantar flexion in humans. *Journal of Applied Physiology* **119,** 1262–1271 (2015).
7. Chino, K. *et al.* In vivo fascicle behavior of synergistic muscles in concentric and eccentric plantar flexions in humans. *Journal of electromyography and kinesiology : official journal of the International Society of Electrophysiological Kinesiology* **18,** 79–88 (2008).
